# Spontaneous Hepatic Hydatid Cyst Rupture Into the Transverse Colon

**DOI:** 10.7759/cureus.16799

**Published:** 2021-07-31

**Authors:** Sadhasivam Ramasamy, Pranav M Singhal, Manu Vats, Sushanto Neogi

**Affiliations:** 1 General Surgery, Milton Keynes University Hospital, Milton Keynes, GBR; 2 Surgical Oncology, Sawai Man Singh Medical College, Jaipur, IND; 3 General Surgery, Maulana Azad Medical College, New Delhi, IND

**Keywords:** echinococcus granulosus, transverse colon, parasitic disease, general surgery, metastatic hydatidosis

## Abstract

Hydatid disease is a prevalent parasitic infestation caused by the cestode *Echinococcus granulosus* in predominantly rural areas of the Mediterranean region, South East Asia, Australia, and South America. This report discusses a unique case of a 32-year-old lady who presented to the Emergency Surgery Department with complaints of abdominal pain, distension, and constipation for five days. Radiological investigations showed air-fluid levels within a large cyst originating from the liver. Surgical exploration revealed a large hepatic hydatid cyst communicating with the transverse colon with the presence of multiple peritoneal hydatid cysts. Evacuation of the cyst contents, lavage, and excision of the rest of the hydatid cysts was done. Dense adhesions were present involving the liver, large bowel, and duodenum. Therefore, a terminal ileum diversion loop ileostomy was made. Ileostomy was reversed after checking the large bowel anatomy with a distal loopogram.

## Introduction

The infestation with the cestode,* Echinococcus granulosus*, is a significant health problem in places where animal husbandry is common [1]. Though the disease is more rampant in the Mediterranean region, Australia and South America; cases can be seen globally because of increased migration of populations [2,3]. Hydatid disease commonly affects the liver and less frequently the spleen, the lung, and the brain. Liver hydatidosis presents with mechanical complications, such as obstructive jaundice, portal hypertension, and Budd-Chiari syndrome and infection of the cyst causes a hepatic abscess. Hepatic hydatid cyst can cause dissemination or anaphylaxis when a cyst ruptures into the biliary tract or peritoneum [4]. The disease is also endemic in India, where the annual incidence ranges from 1 to 200 per 100,000 population [5]. Hydatid cysts of the liver may rupture into the biliary channels, pleural cavity, pericardial cavity, or become infected with bacteria [6]. Direct perforation of the cyst into hollow abdominal organs is a very unusual occurrence. Nevertheless, communication of the cyst with the duodenum or the stomach has been reported in the literature [7-9]. Rupture of a hydatid cyst into the colon is extremely rare and only a few cases have been reported till now [10-14].

## Case presentation

A 32-year-old lady presented to the Emergency Surgery Department, Lok Nayak Hospital, New Delhi with complaints of generalized abdominal pain, distension, vomiting, and constipation for five days and fever for two days. There was no history of any similar complaint in the past. There was no history of the passage of any mucoid material in the vomitus or the stools. There was no history of any prior hospital admission for any surgical procedure. She did not suffer from diabetes mellitus, hypertension, or tuberculosis. The patient was a non-smoker, non-alcoholic and did not chew tobacco. She had normal menstrual cycles. The family history was non-contributory.

On examination, she was dehydrated, and her vitals were stable. The abdomen was distended and bowel sounds were sluggish. However, there were no signs of peritonitis.

**Figure 1:**
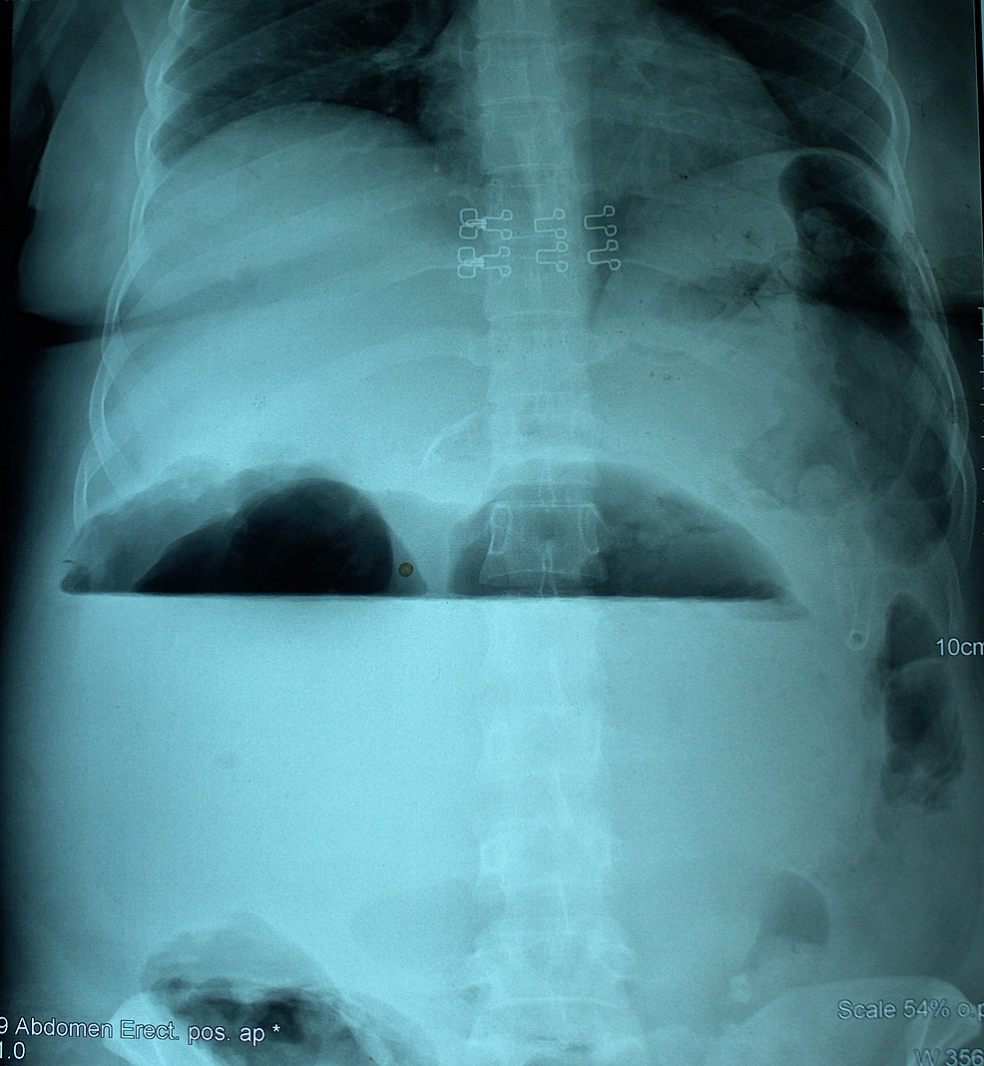
Plain abdominal x-ray showing a large air-fluid level within the hepatic hydatid cyst.

The full blood count (FBC) of the patient showed a white blood cell (WBC) count of 12,000 with neutrophilia (75%), eosinophilia (15%), hemoglobin of 8.6 gm/dL, and normal platelet counts. The renal and liver function tests were normal. The coagulation profile was normal. Enzyme-linked immunosorbent assay (ELISA) for anti-*Echinococcus* antibodies was positive. X-ray abdomen (erect) (Figure 1) showed a large air-fluid level in the upper abdomen.

**Figure 2:**
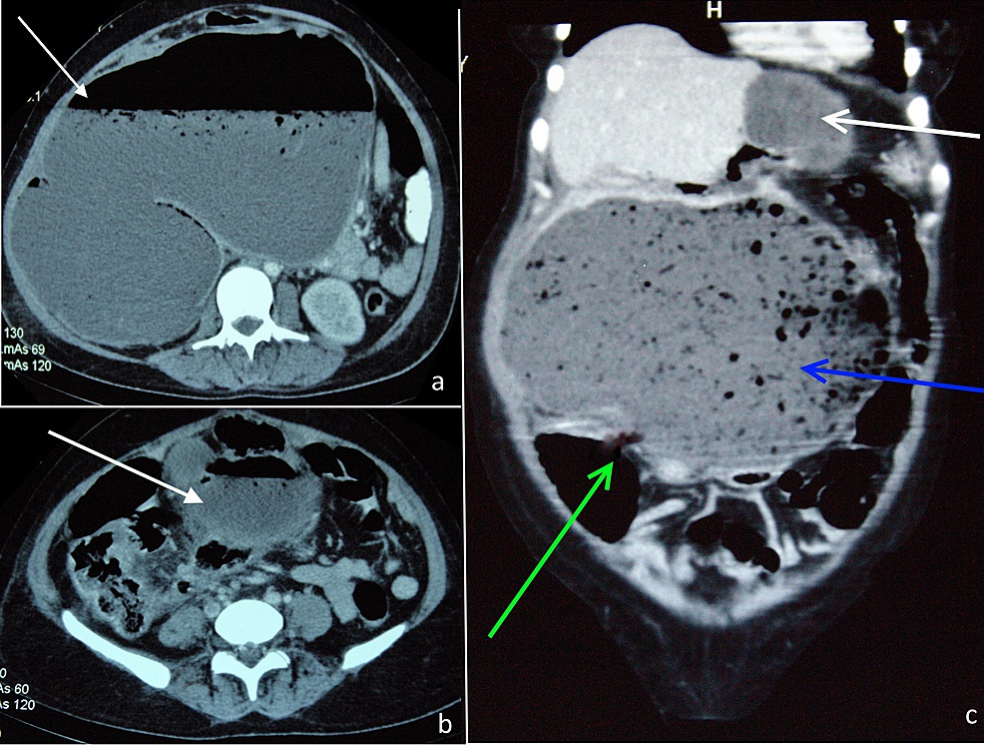
CECT showing multiple hydatid cyst in the (a) liver and (b) omentum (white arrow). (c) Coronal view showing large hydatid cyst from liver (blue arrow) ruptured into the colon (green arrow). CECT - Contrast-enhanced computed tomography

Requisition for a contrast-enhanced computed tomography (CECT) abdomen was sent to the Radiology Department and the patient was scheduled for the scan after one week. However, on day 5 of admission, the patient developed tachycardia and severe abdominal pain. The abdomen showed signs of localized peritonitis in the right hypochondrium, epigastrium, right lumbar and umbilical segments. After discussing the case with the radiologist, the patient was shifted for an urgent CECT abdomen. The scan showed a large cystic lesion with air-fluid level arising from the liver with probable rupture of cyst into the colon. Multiple hydatid cysts in the liver, omentum, and pelvis were noted (Figures 2a-2c).

**Figure 3:**
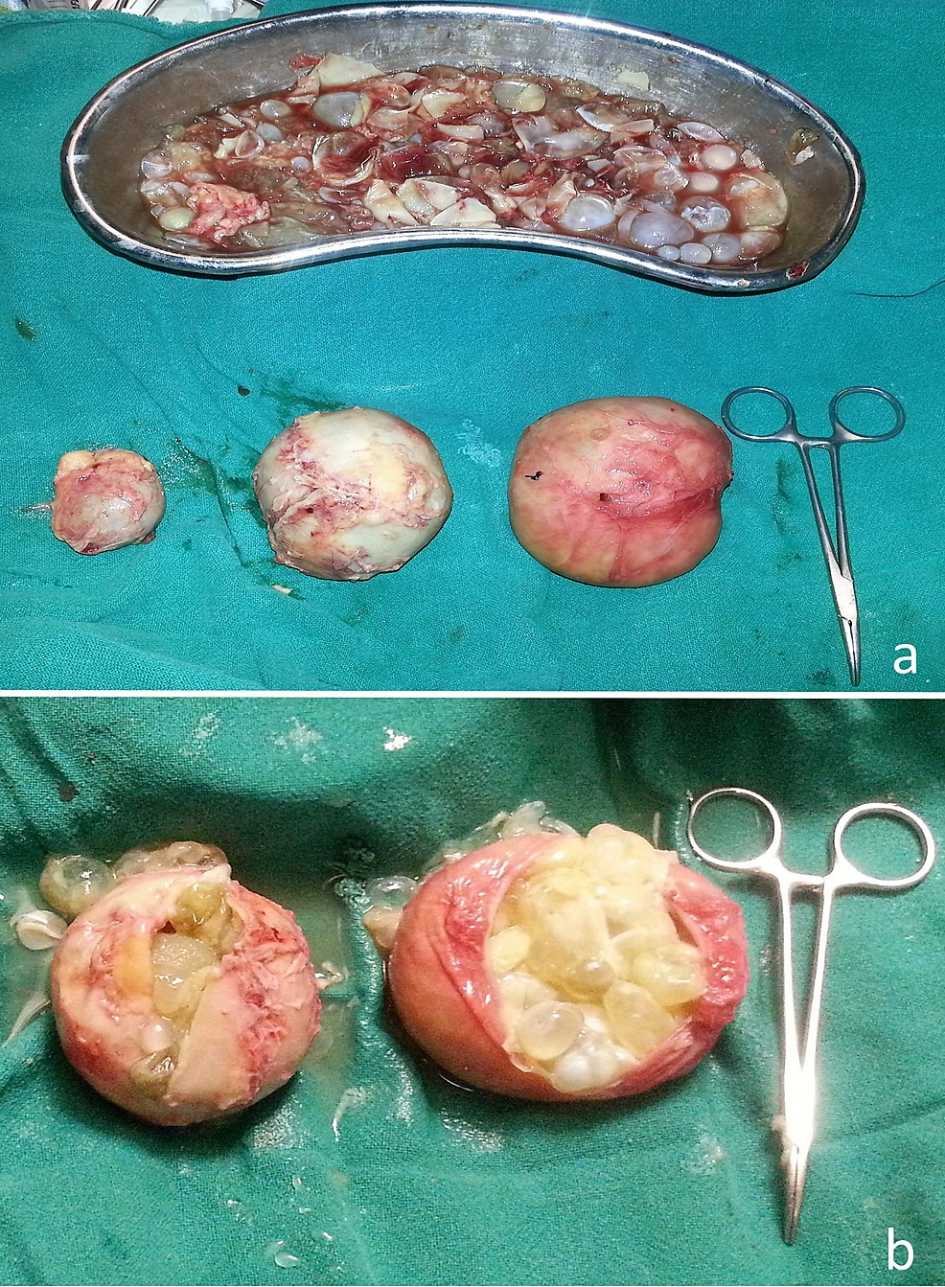
(a) Excised hepatic, omental, and pelvic hydatid cyst. (b) Multiple daughter cysts within the specimen.

The patient underwent an exploratory laparotomy. Intraoperatively, a cystic lesion of 12×7×9 cm was seen in the right lobe of the liver, filled with daughter cysts. The cyst was isolated with sponges soaked in the povidone-iodine solution. The cyst densely adhered to the hepatic flexure of the colon with minimal fecal content within the cyst cavity. However, it was extremely difficult to delineate and dissect out the fistulous tract due to dense inter-bowel and hepato-colic adhesions. The entire contents of the liver cyst cavity were evacuated, and thorough lavage was performed. A drain was placed within the cavity thereafter. Keeping in mind the high likelihood of occurrence of iatrogenic injuries and hemodynamic instability of the patient, it was decided not to proceed further with adhesiolysis. The remaining hepatic, omental and pelvic hydatid cysts (Figures 3a, 3b) were excised completely and a temporary diversion ileostomy was made, with a plan to evaluate and assess the colon anatomy at a later date.

**Figure 4:**
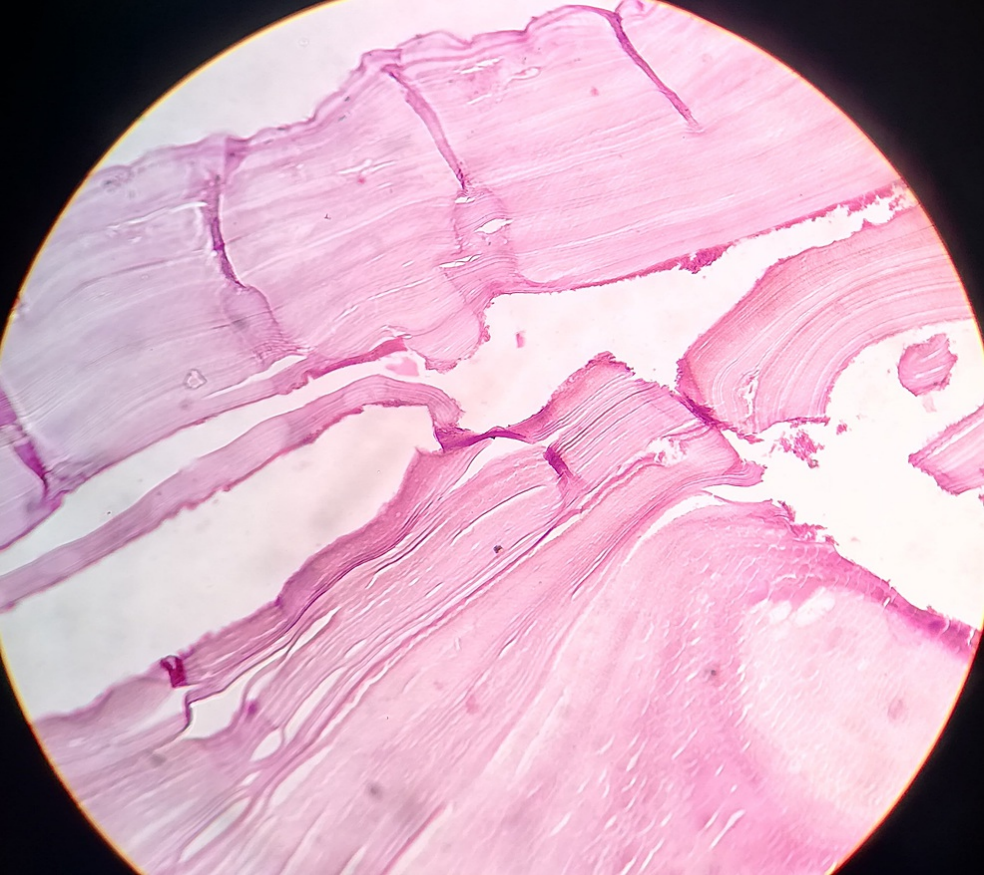
Photomicrograph of the hydatid cyst showing the outer acellular laminated membrane and inner nucleate germinal layer (H&E stain 50x).

The post-operative period was uneventful, and the cyst cavity drain was removed on the 13th post-operative day when the output was reduced to nil. Histopathology report confirmed the diagnosis of hydatid disease in the liver, omentum, and pelvis (Figure 4).

The patient was discharged thereafter; with a plan to close the diversion ileostomy after a contrast distal loopogram study for confirming the absence of any fistulous communication between the colon and liver or peritoneal cavity. She was discharged on the 14th postoperative day after fully tolerating an oral diet. She was prescribed tablet albendazole 400 mg BID on discharge and was advised to follow up on a regular basis. Distal loopogram after six weeks did not reveal any fistulous communication of colon to the liver or peritoneal cavity. The patient underwent ileostomy closure after three months of the index operation. She made an uneventful recovery and was doing well at the end of three months follow-up.

## Discussion

Clinically, the hydatid cyst rupture into a hollow organ can be diagnosed by the passage of hydatid membranes in the stools (hydatidorrhea or hydatidenteria) or the vomitus (hydatidemesis) [14]. However, a patient can also present with non-specific signs, such as abdominal discomfort, lump or pain, dyspepsia, or fever [15]. Hydatid disease can usually be diagnosed preoperatively with ultrasonography (US) and CT. The US is a widely used radiological modality to diagnose hydatid disease. Hydatid disease in its initial or active phase may appear as an anechoic, well-demarcated cystic lesion with small echogenic foci (falling snowflakes), which appear as hydatid sand and change with patient position. CT shows a water attenuating lesion with clearly defined borders. Host tissue compression shows pseudo contrast enhancement of the cyst wall. However, these findings change as the disease progresses and every stage shows characteristic ultrasonographic and CT features [16]. 

Several diseases may have a similar clinical presentation to the case discussed herein. These include ruptured amoebic or pyogenic liver abscess, ruptured hydatid cyst, abdominal abscess, intestinal duplication cyst, and mesenteric cyst.

 Three types of hydatid cyst rupture have been described in the literature: contained, communicating, and direct. When the endocyst ruptures and the fluid seeps through space in between the pericyst and endocyst resulting in the collapse of the endocyst, it is known as a contained rupture. Communicating rupture refers to a rupture into the biliary tree and is the most common variant [17]. Rupture of the cyst contents directly into the peritoneal cavity, mediastinum, thorax, or gastrointestinal tract is known as direct rupture [18]. If the cyst ruptures directly into the peritoneal cavity, implantation of scolices may occur in multiple organs. This condition is appropriately termed “metastatic hydatidosis” [19]. The present case is an example depicting direct rupture. The cyst probably ruptured earlier into the peritoneal cavity which led to implantation of scolices and resulted in multiple peritoneal hydatid cysts. Presently, the cyst has ruptured directly into the transverse colon.

Two mechanisms have been suggested, which may be responsible for the rupture of a hydatid cyst into the surrounding structures. Infection of the cyst content and swelling of the outer membranes result in adhesions between the cyst and surrounding viscera. Continuous mechanical friction by the pericyst can result in erosion into the hollow viscus wall [18]. It was difficult to ascertain intra-operatively as to which mechanism was responsible for the rupture of the cyst into the transverse colon in our case. However, the presence of dense adhesions may suggest that infection of the cyst could have been the cause.

There are only a very limited number of reports describing hydatid cyst rupture into the gastrointestinal tract. Hepatic hydatid cysts fistulizing into the duodenum have been described in English literature. Patients were managed by surgical exploration and made successful recovery [7]. Rupture of a hydatid cyst into the colon is extremely rare and only six cases have been reported till now (Table 1).

**Table 1:** A literature review of cases describing the rupture of a hydatid cyst into the colon

Authors	Year	Case report
Morris et al. [11]	1983	Colohepatic fistula due to hydatid disease
Lo Casto et al. [9]	1997	Hydatid cyst of the liver communicating with the left colon
Teke et al. [2]	2008	Splenic hydatid cyst perforating into the colon manifesting as acute massive lower gastrointestinal bleeding: an unusual presentation of disseminated abdominal echinococcosis
Bougioukas et al. [10]	2009	Liver hydatid cyst perforated into the large bowel
Chattopadhyay et al. [12]	2012	Hydatid cyst-colonic fistula: endogenic with ectogenic vesiculation
Garg et al. [13]	2018	Isolated hydatid cyst of spleen with cystocolic fistula: an unusual case

A report describes perforation of a hepatic hydatid cyst into the right colon, which was managed by partial cystectomy, omentoplasty, right hemicolectomy, and side-to-side ileocolic anastomosis. The patient had an uneventful recovery [10]. Casto et al. described a case of hepatic hydatid cyst rupturing into the left colon which was managed by cyst drainage, partial cystectomy, and suture of the colonic fistula [9]. In an exhaustive study by Kourias et al., out of the 1,296 hepatic hydatid cysts operated, only seven showed communication with the gastrointestinal tract (two in the stomach, two into the duodenum, and three into the colon) [20]. According to research, only 13 cases of hepatic hydatid cysts communicating with the duodenum have been described [6].

Treatment following rupture of a hydatid cyst is always surgical. Total resection of the cysts is the treatment of choice. If that is not possible, then cyst enucleation, deroofing of the cyst with omentoplasty or external drainage may be done [20]. Free intraperitoneal hydatid cysts can be easily resected as they usually do not adhere firmly to other organs [2]. In cases of rupture into hollow viscus, the surgical procedure depends on the extent of hollow viscus damage and includes excision of a fistula between the viscus and the cyst with the repair of the hollow organ with medical management for the parasite.

## Conclusions

Hepatic hydatid cyst rupturing into the transverse colon is a very rare complication of hydatid disease. A patient with a ruptured hydatid cyst may present with a variety of signs and symptoms, ranging from dull aching pain and mild abdominal distension to peritonitis. A pre-operative diagnosis of cyst rupture into the gastrointestinal tract can be supported with the aid of the CECT abdomen showing the characteristic findings. Surgical exploration is the definitive treatment of a ruptured hydatid cyst and is usually followed by a good prognosis.
